# Integration of Nonlinear Rheology and CFD Simulation to Elucidate the Influence of Saturated Oil on Soy Protein Concentrate Behavior During High-Moisture Extrusion

**DOI:** 10.3390/gels11121003

**Published:** 2025-12-12

**Authors:** Timilehin Martins Oyinloye, Chae-Jin Lee, Won Byong Yoon

**Affiliations:** 1 Department of Food Science and Biotechnology, College of Agriculture and Life Sciences, Kangwon National University, Chuncheon 24341, Republic of Korea; oyinloyetm@kangwon.ac.kr (T.M.O.); 202515177@kangwon.ac.kr (C.-J.L.); 2 Elder-Friendly Food Research Center, Agriculture and Life Science Research Institute, Kangwon National University, Chuncheon 24341, Republic of Korea; 3 Department of Food Biotechnology and Environmental Science, Kangwon National University, Chuncheon 24341, Republic of Korea

**Keywords:** alternative meat, soy protein gelation, co-extrusion, gelation kinetics, oil diffusion, process simulation, extrusion optimization, LAOS, SAOS

## Abstract

This study investigated the influence of coconut oil concentration (0–2%) on the nonlinear rheological and thermal behavior of soy protein concentrate (SPC) mixtures and integrated these data into computational fluid dynamics (CFD) models to predict flow behavior during high-moisture extrusion. Temperature sweep tests revealed that increasing oil content elevated the onset and peak gelation temperatures from 64.13 to 70.21 °C and 70.29 to 76.08 °C, respectively, while decreasing gelation enthalpy from 4.05 J/g to 2.81 J/g. Large-amplitude oscillatory shear (LAOS) analysis showed a shift from strain-stiffening (e_3_/e_1_ > 0.15) behavior to strain-thinning (e_3_/e_1_ < 0.05) behavior with increasing oil, accompanied by enhanced shear-thinning behavior (v_3_/v_1_ < 0). Integrating these nonlinear parameters into the CFD simulations enhanced model accuracy relative to the SAOS-based approach, resulting in lower RMSE values (≤4.41 kPa for pressure and ≤0.11 mm/s for velocity) and enabling more realistic prediction of deformation and flow under extrusion-relevant conditions, a capability that conventional SAOS-based models could not achieve. Predicted outlet melt temperatures averaged 70.27 ± 1.55 °C, consistent with experimental results. The findings demonstrate that oil addition modulates protein network formation and flow resistance, and that nonlinear rheology-coupled CFD models enable reliable prediction of extrusion behavior. Overall, this study provides a novel rheology-driven modeling strategy for enhancing the design and optimization of oil-enriched plant-protein extrusion processes.

## 1. Introduction

The rapid growth of plant-based meat analogs relies on mimicking the complex texture, juiciness, and sensory profile of animal muscle [1,2]. Soy protein concentrate (SPC) is widely favored for its functional properties and ability to form fibrous structures during thermomechanical processing [3,4]. However, reproducing meat-like texture requires fine control over the protein–moisture–lipid interactions within the High-Moisture Extrusion (HME) process [1,2].

HME subjects a hydrated protein blend to intense mixing, shearing, and thermal denaturation, transforming it from a paste into a viscoelastic melt, which is subsequently aligned and solidified into an anisotropic weak gel structure upon cooling [2,5]. The rheological properties of this melt, particularly its viscosity and viscoelastic moduli, govern the material flow and molecular alignment, directly dictating the final product quality, mechanical strength, and fibrousness [6]. Therefore, controlling the rheology of the soy-protein melt across these thermal-mechanical states is crucial for effective product design.

Among the key formulation factors, lipids (such as saturated coconut oil) are essential for enhancing sensory attributes like juiciness and mouthfeel [7,8]. While several studies show that oil inclusion reduces torque and die pressure, and alters strand expansion [9,10], the precise mechanisms by which saturated oils modify the rheological and structural evolution of soy-protein melts remain incompletely understood. The effect of oil is complex and state-dependent: it can act as a lubricant in the paste and melt states, reducing viscosity and elasticity by interrupting protein associations [11]. Conversely, upon cooling, solidified saturated oil droplets may act as rigid fillers or reinforcement agents, potentially increasing the stiffness of the final gel network [11,12].

To thoroughly investigate this interplay, detailed rheological characterization is necessary. While Small-Amplitude Oscillatory Shear (SAOS) tests provide insight into the linear viscoelastic domain [11], the high-strain, high-shear environment of extrusion requires Large-Amplitude Oscillatory Shear (LAOS) analysis to capture nonlinear viscoelasticity, including yielding, structural breakdown, and strain-softening behavior [13]. Furthermore, rheological data form the foundation for Computational Fluid Dynamics (CFD) modeling [11,14]. Integrating rheological constitutive models, especially those reflecting nonlinear LAOS behavior, into CFD simulations is essential for accurately predicting key process variables (e.g., pressure, velocity, and temperature) and virtually optimizing flow and alignment within the cooling die [15,16].

Previous research in soy-protein systems has primarily focused on the influence of protein type, moisture content, or general process parameters [6,17,18]. Comparatively fewer works have systematically examined the specific, concentration-dependent role of saturated oil on the viscoelastic properties across the paste, melt, and weak-gel states. Moreover, the critical coupling of nonlinear (LAOS) rheology with CFD modeling to predict the extrusion flow behavior of oil-enriched systems is underexplored. A detailed investigation of these effects will clarify whether changes in oil content primarily influence the linear flow regime or induce significant nonlinear yielding behavior, providing information crucial for scaling formulations to industrial practice.

Therefore, the present study aims to investigate the influence of saturated coconut oil concentration (0–2%) on the rheological properties and flow characteristics of soy-protein concentrate during high-moisture extrusion. Specifically, this study seeks to: (1) characterize the effects of incremental oil levels on SPC rheology across the paste, hot-melt, and weak-gel states using SAOA; (2) quantify the nonlinear (LAOS) viscoelastic response to determine how oil modifies yielding and strain-dependent structural behavior; (3) develop CFD models incorporating SAOS- and LAOS-based constitutive parameters to predict pressure, velocity, and thermal profiles under extrusion-relevant conditions; and (4) evaluate whether integrating nonlinear rheology into CFD improves predictive accuracy compared with traditional SAOS-based modeling. Ultimately, this work aims to provide a scientifically grounded pathway for optimizing extrusion formulation and processing strategies in oil-enriched plant-protein systems.

## 2. Results and Discussion

### 2.1. Linear Rheological Behavior of SPC Mixture

#### 2.1.1. Steady-Shear Viscosity Behavior of SPC Mixtures with Varying Oil Content

**Figure 1:**
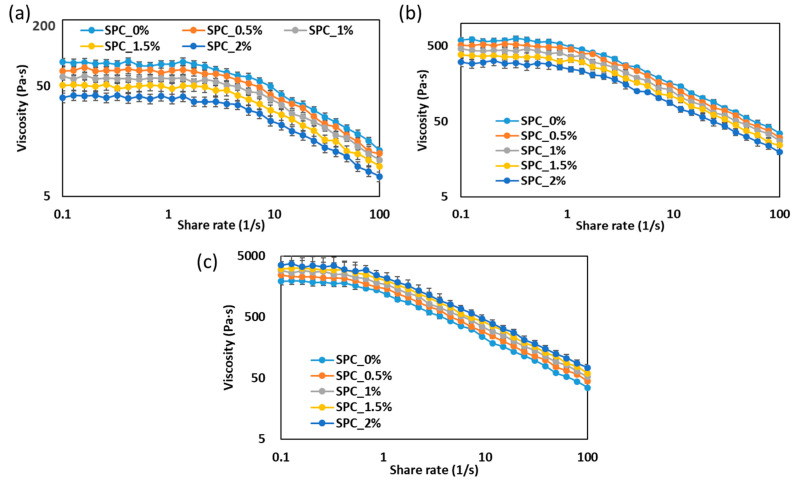
Apparent viscosity of SPC mixtures with different oil concentrations measured at three thermal states: (**a**) paste at 25 °C, (**b**) molten melt at 100 °C, and (**c**) weak gel at 70 °C. Error bars indicate ± standard deviation.

The steady-shear viscosity profiles reveal distinct thermomechanical behaviors across the three structural states of SPC with increasing oil content (Figure 1). In the paste (at 25 °C, Figure 1a) and molten state (at 25 °C, Figure 1b), apparent viscosity decreases monotonically with increasing oil concentration, consistent with oil acting as a lubricating phase that interrupts protein–protein contacts and lowers intermolecular friction during flow. This lubricating effect and the resulting reduction in shear resistance are commonly reported outcomes when lipids are introduced into protein pastes or melts [9,18].

By contrast, in the partially gelled state (weak-gel state, 70 °C, Figure 1c), the trend reverses: samples with higher oil (notably SPC_2%) exhibit the highest viscosities while the oil-free sample (SPC_0%) is the least viscous. This inversion indicates that during thermal restructuring and network formation, the dispersed oil phase transitions from an essentially “inert” lubricant to an active structural modifier. Several mechanisms can explain this behavior. First, protein adsorption at oil–water interfaces can create interfacial protein films that couple droplets to the gel matrix; these coated droplets behave as reinforcing inclusions (active fillers), increasing resistance to deformation [19]. Secondly, oil droplets can promote local concentration or bridging of protein aggregates during cooling, effectively increasing network density and bulk viscosity [20]. Thirdly, droplet size and interfacial properties determine whether droplets act as stress-bearing entities or void-like defects; well-stabilized, small droplets typically strengthen gels while poorly stabilized or coalesced droplets weaken them [19].

Taken together, the state-dependent trends, viscosity decrease with oil in flowing (paste/melt) states and viscosity increase with oil in the cooling/weak gel state, are consistent with the dual role of oil reported in emulsion-filled gel literature: lubrication during flow and filler-like reinforcement during gelation when droplets are coupled to the matrix [21]. These results emphasize that the functional effect of coconut oil depends critically on the thermal/mechanical history (i.e., whether the sample is above or below the temperature window where protein aggregation and network formation dominate) and on droplet interfacial characteristics. For process design, this implies that small oil additions may improve processability in the melt but simultaneously alter final gel strength and texture after cooling; an effect that should be controlled via droplet size, emulsification strategy and cooling profile.

#### 2.1.2. Strain Sweep Analysis of SPC with Different Oil Content

**Figure 2:**
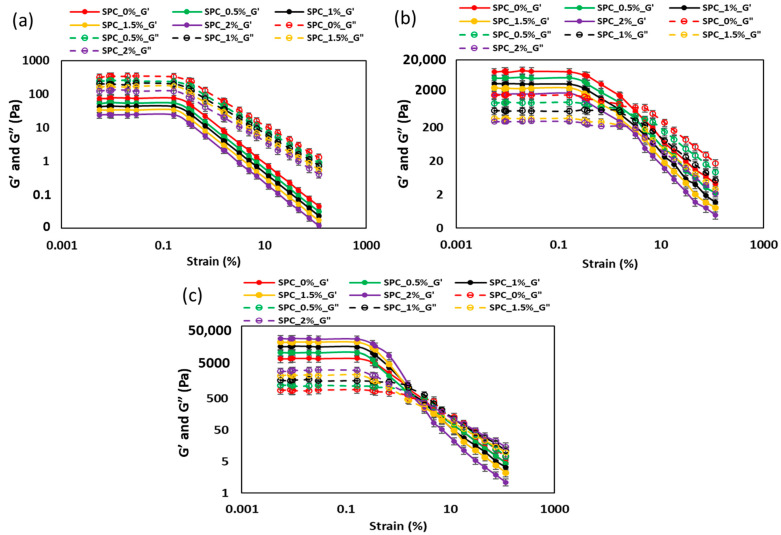
Strain sweep results (G′ and G″) for SPC mixtures containing 0–2% oil measured in three material states: (**a**) paste (25 °C), (**b**) molten melt (100 °C), and (**c**) weak gel (70 °C). Error bars indicate ± standard deviation.

The strain-sweep data for SPC with varying oil contents show markedly different behavior across three structural states: paste (25 °C), molten (100 °C) and weak-gel (70 °C) (Figure 2, Table 1). In the paste state, the loss modulus (G″) remains higher than the storage modulus (G′) over the full strain range and no crossover strain was observed, reflecting a viscous-dominated system lacking a continuous elastic network (Table 1). All moduli decline with increasing oil concentration, indicative of oil acting as a lubricant or diluent that disrupts protein–protein linkages (e.g., hydrogen bond, hydrophobic interactions) and lowers network connectivity.

Upon heating to the molten state, G′ surpasses G″, and a crossover point is observed (Figure 2): both the crossover strain and the critical strain (γ_cr_) decrease as oil concentration increases. Simultaneously, G′ at γ_cr_ and cohesive energy density decline steadily from 0% to 2% oil. This pattern suggests that while heat denaturation enables network formation, added oil weakens the emerging structure, likely through interference in protein aggregation or by introducing droplet lubrication between unfolding protein chains. Similar weakening trends with oil have been documented in plant-protein emulsion systems [22].

In the weak-gel state (cooled to 70 °C), the system behaves as an elastic network: G′ remains significantly higher than G″, and notably increases with oil content (from ~9500 Pa at 0% oil to ~36,500 Pa at 2% oil), with no significant difference between SPC_1.5% and SPC_2%. Cohesive energy density mirrors this trend, peaking at ~1715 J m^−3^ at 1.5% oil. This reversal, i.e., oil acting to strengthen rather than weaken, aligns with the concept of “active-filler” droplets: when oil droplets are well integrated into a protein matrix via interfacial adsorption, and they act as reinforcing particles, enhancing the gel network stiffness [22,23]. In the SPC system, the plateauing of G′ at 1.5% oil suggests a saturation of droplet–matrix coupling or droplet overcrowding, which prevents further stiffening.

Chemically, this heat- and oil-dependent behavior reflects a balance of competing effects. During flow, protein network build-up (denaturation, aggregation, and cross-linking) is countered by oil droplet interference and lubrication. Conversely, during gelation, the solidified oil droplets become embedded, leading to network reinforcement. The dual role of oil, reducing viscosity and network strength during flow (paste/melt) while enhancing elastic rigidity after gelation (weak gel), has significant implications for high-moisture extrusion processes: oil content must be tuned to optimize both processability and final texture. These findings support more general formulations of emulsion-filled gel behavior, where droplet–matrix interaction, interfacial chemistry, and structural state govern rheology [24].

#### 2.1.3. Effect of Oil Content on the Frequency-Dependent Viscoelastic Behavior of SPC Mixture

**Figure 3:**
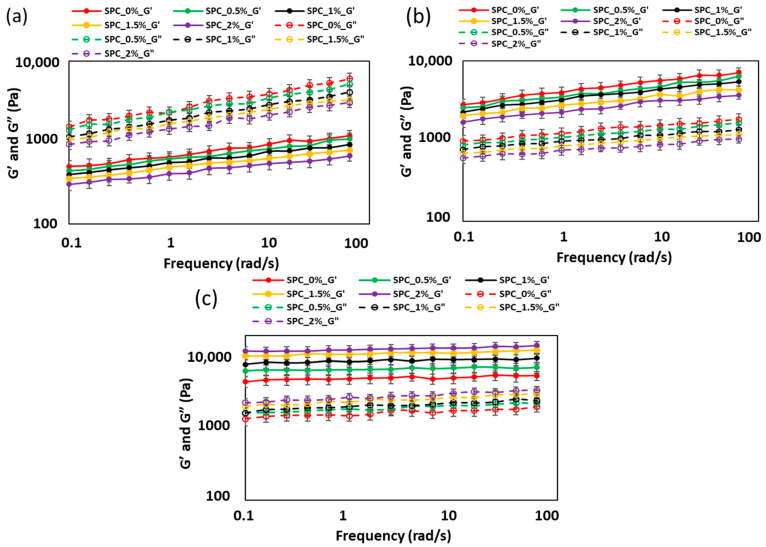
Dynamic frequency sweep curves of SPC mixtures at different oil concentrations: (**a**) paste state, (**b**) molten state, and (**c**) weak-gel state. The solid and dashed lines represent the storage (G′) and loss (G″) moduli, respectively, as a function of angular frequency (0.1–100 rad/s). Error bars indicate ± standard deviation.

The frequency sweep results presented in Figure 3 and the viscoelastic parameters summarized in Table 2 describe how the mechanical spectra of SPC mixtures evolved with oil concentration and physical state. These data provide valuable insight into how the system transitions from a viscous paste to an elastic melt and finally to a more structured weak gel during cooling.

In the paste state, the damping factor (G″/G′) values at 1–100 rad/s were between 3.3 and 5.0, clearly indicating viscous-dominated behavior. Both K′ and K″ decreased steadily with oil addition, from 679.3 Pa·s^n^ (SPC_0%) to 411.2 Pa·s^n^ (SPC_2%), while the flow exponents (n′ and n″) also dropped slightly, suggesting that oil weakened intermolecular associations within the protein matrix. This reduction in viscoelasticity is attributed to oil interrupting hydrophobic and hydrogen bonds that normally promote network formation between protein molecules. Similar weakening effects were reported for a study on soy protein isolates containing castor oil and glycerol blends. The authors observed that the incorporation of small amounts of castor oil into glycerol-plasticized soy protein plastics led to a homogeneous dispersion, but at higher concentrations, phase separation occurred, reducing G′ and G″ due to diminished protein–protein interactions [25].

At the molten state (100 °C), the damping factor dropped to around 0.26–0.34, showing that the material had transitioned to an elastic-dominated response. This reflects protein unfolding and aggregation under heat, forming a semi-continuous viscoelastic network. However, K′ and K″ still decreased as oil concentration increased (K′: 4071 to 2334 Pa·s^n^; K″: 1254 to 746 Pa·s^n^), suggesting that the oil interfered with intermolecular crosslinking during thermal denaturation. Xu et al. [26] review of protein-stabilized emulsion gels highlights how interfacial and bulk interactions control whether droplets weaken or disrupt developing protein networks under thermal/mechanical stress.

Interestingly, in the weak-gel state, the trend reversed: K′ and K″ increased with oil content (K′: 4962 to 12641 Pa·s^n^; K″: 1598 to 2667 Pa·s^n^), while the damping factor decreased from 0.30 to 0.22. This suggests that oil droplets acted as fillers or reinforcement agents within the protein network during cooling, possibly due to interfacial protein adsorption and partial coalescence that improved network continuity. The increase in elastic strength up to 1.5% oil also aligns with a saturation point where additional oil no longer significantly enhances structure.

Overall, the frequency sweep results indicate a state-dependent effect of coconut oil: it reduces elasticity in fluid states (paste and melt) but enhances network stiffness during gelation. This behavior underscores the dual role of oil in modulating both flowability during extrusion and textural integrity upon cooling, a key factor for optimizing plant-based meat analogs.

### 2.2. Thermal Kinetics of Gelation in SPC Mixtures

**Figure 4:**
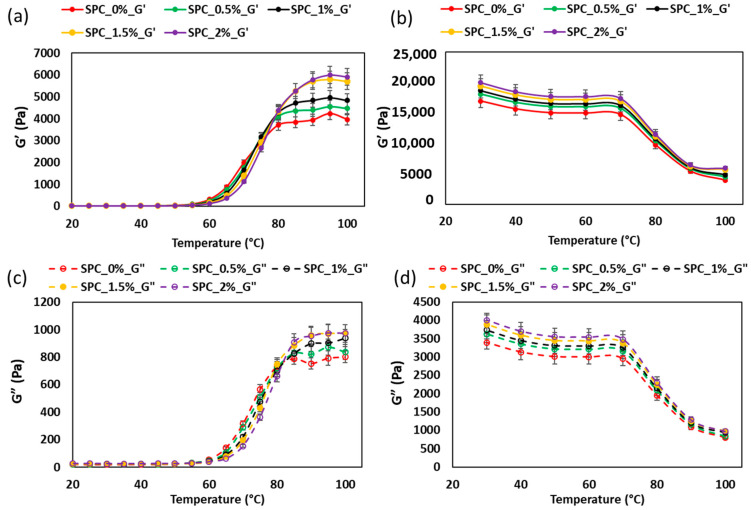
Temperature sweep rheology of SPC mixtures (0–2% coconut oil): (**a**) storage modulus G′ during heating, (**b**) G′ during cooling, (**c**) loss modulus G″ during heating, and (**d**) G″ during cooling. Error bars indicate ± standard deviation.

**Figure 5:**
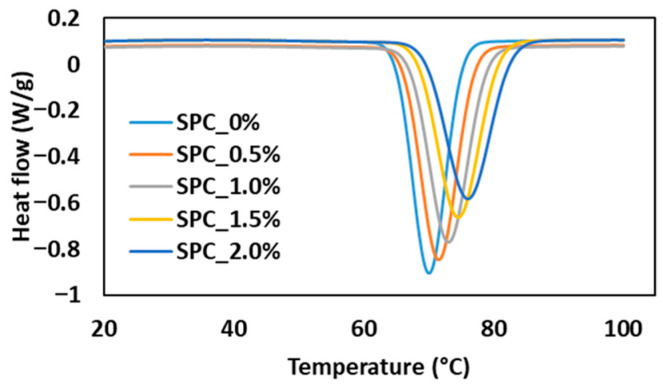
DSC thermograms of SPC mixtures with varying oil concentration, showing endothermic transitions associated with protein gelation/aggregation during heating.

The temperature sweep rheological profiles (Figure 4) and DSC data (Figure 5, Table 3) provide complementary insight into the gelation kinetics of SPC–oil blends. Across all samples, incorporation of oil shifted the onset, peak and end-gelation temperatures to higher values: onset rose from 64.1 °C at 0% oil to 70.2 °C at 2% oil, peak from 70.3 °C to 76.1 °C, and end set temperature from 76.3 °C to 82.7 °C. Similarly, the gelation enthalpy initially increased at low oil levels (4.05 J/g at 1.5% oil) before dropping at higher oil levels (3.91 J/g at 2%).

During the heating phase (Figure 4a,c), G′ and G″ remained low until the onset temperature, then rose sharply, reflecting aggregation, unfolding, and network formation of the soy proteins, consistent with the classic gelation behavior described for soy protein gels [27]. The upward shift of gelation temperature with increasing oil suggests that the oil phase delays protein–protein association, likely by increasing local viscosity, reducing effective collision frequency of denatured protein domains, or by acting as an inert phase that must be excluded from the network before percolation.

In the cooling phase (Figure 4b,d), G′ and G″ of all samples stabilized into the weak-gel state at 71 ± 1.35 °C, the temperature selected as the target for the extrudate. Notably, a distinct reversal in G′ and G″ trends occurred around 70 °C: for the 2% oil sample, G′ increased by approximately 12.23% during this transition, while G″ decreased by 13.66%. Lower-oil samples (0–0.5%) showed a more modest G′ increase of approximately 6.12%, indicating that oil amplifies the solid-like character as the matrix cools. This quantitative shift reflects faster elastic network consolidation in oil-rich formulations once protein–protein interactions dominate over thermal softening. In practical extrusion terms, the sharper rise in G′ at cooling temperatures corresponds to higher melt stiffness, which is typically associated with reductions in screw torque and die pressure during flow because the oil phase acts as a lubricant prior to gel set. However, once gelation proceeds past ~70 °C, the rapid stiffening can increase resistance to deformation in the cooling die, influencing strand alignment and final texture development. The DSC enthalpy decrease at 2% oil supports this interpretation, indicating partial loss of aggregated network structure at excessive oil levels.

Comparisons with literature show that soy-protein gels frequently exhibit gelation onset around 65–70 °C and plateau moduli that depend on pre-heating and network density [28]. The present findings emphasize how coconut oil modulates both kinetics (temperature shifts) and final gel strength (moduli, enthalpy) in a state- and concentration-dependent manner. From a processing perspective, the delayed gelation onset and higher required gelation temperature with oil must be accommodated in the cooling die design to ensure full network development before the product is extruded and final texture formation.

### 2.3. Analysis of Normalized Lissajous-Bowditch Curves for SPC Mixtures

**Figure 6:**
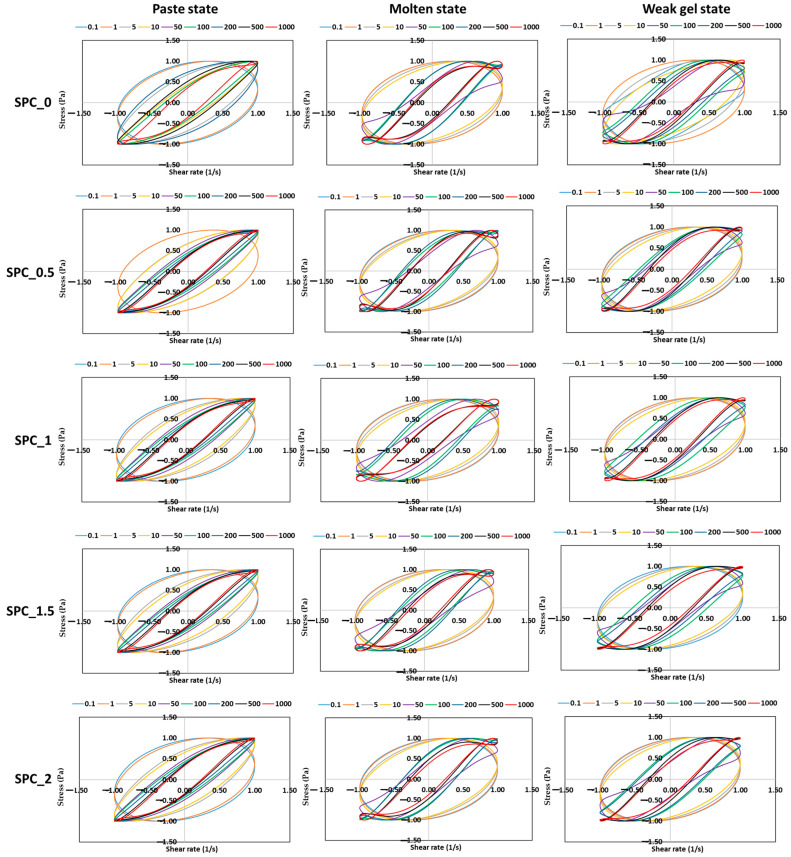
Elastic Lissajous–Bowditch curves (normalized stress–strain) of SPC mixtures with 0–2% coconut oil under large-amplitude oscillatory shear at 1 rad s^−1^. Columns correspond to paste, molten, and weak-gel states; rows represent oil concentration. Strain amplitudes range from 0.1% to 1000%.

**Figure 7:**
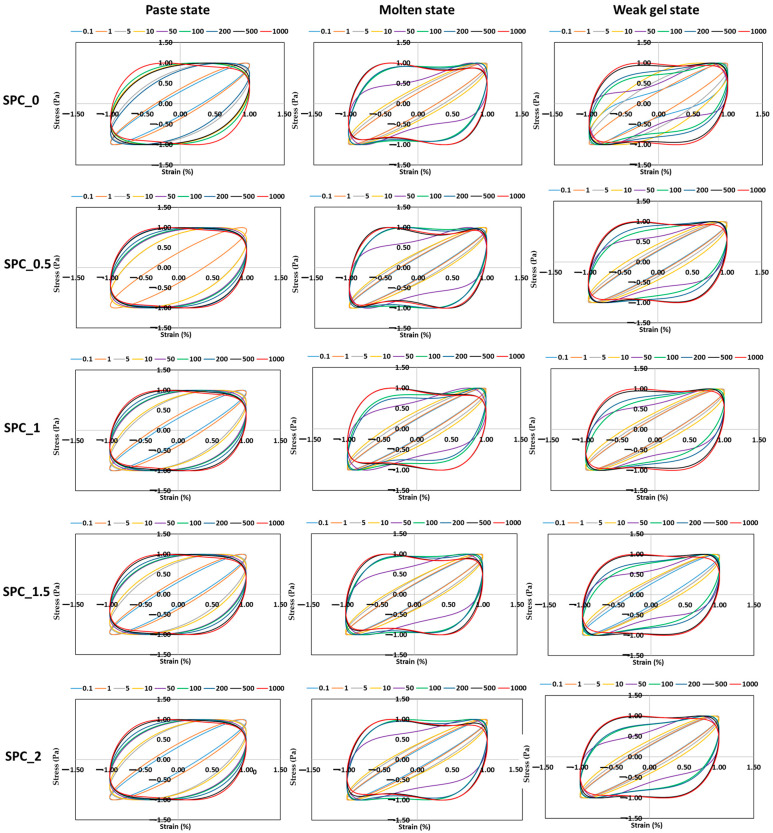
Viscous Lissajous–Bowditch curves (normalized stress–strain-rate) of SPC mixtures with 0–2% coconut oil at 1 rad s^−1^. Loop distortion illustrates the evolution of nonlinear viscous behavior across strain amplitudes and thermal states.

LAOS tests were conducted to assess the SPC mixture’s nonlinear rheological behavior under large deformations. Figure 6 shows the elastic Lissajous–Bowditch (L-B) curves (Stress Vs. Strain) for SPC mixtures containing 0–2% oil across the paste, molten, and weak-gel states. The corresponding viscous L-B plots (Stress Vs. Strain-Rate) are presented in Figure 7. Each subplot captures intracycle nonlinear viscoelastic behavior at increasing strain amplitudes (0.1–1000%), illustrating how the internal microstructure evolves under large deformation.

At low strains (≤1%), all samples exhibited nearly elliptical elastic loops, typical of the linear viscoelastic regime where stress is proportional to strain and dominated by reversible elastic deformation. As strain increased (~5%), curve distortion became apparent, reflecting the onset of nonlinear viscoelasticity as protein–protein and protein–oil interactions were disrupted. In the paste state, the L–B curves were broader for lower oil contents (SPC 0 and 0.5%), showing higher resistance to deformation and stronger network rigidity (Figure 6). Increasing oil concentration caused the loops to become narrower and more elliptical, signifying reduced elastic energy storage and increased molecular slippage due to the oil’s lubricating action. This softening effect has also been observed for oil-enriched soy protein gels and other emulsified protein systems, where dispersed lipid droplets interfere with protein–protein connectivity and weaken interchain bonding [29].

In the molten state, elastic loops remained more elliptical overall, but their slopes increased, indicating greater elasticity as the temperature-induced unfolding of soy globulins enabled entanglement and alignment of protein chains. However, at high strains (≥10%), the curves for all oil levels developed characteristic “S-shaped” distortions, suggesting yielding and partial structural breakdown (Figure 7). The degree of nonlinearity diminished slightly with oil addition, implying that oil reduced cohesive interactions and delayed complete network rupture. These findings align with previous observations indicating that the influence of oil on the rheological properties depends on the material used and whether oil droplets act as an active or inactive filler [30].

For the weak-gel state, the elastic L–B loops exhibited the most pronounced area and sharp corners, particularly at 1.5–2% oil, reflecting a stiffer, energy-storing network dominated by irreversible elastic deformation (Figure 6). This behavior suggests the formation of a reinforced protein–oil network during cooling, where partial crystallization or phase structuring of the coconut oil enhances gel rigidity. Comparable transitions have been described in composite biopolymer gels such as soy-polysaccharide or soy–lipid systems, where oil droplets become embedded in the protein matrix and contribute to the elastic backbone [30].

The viscous L–B curves complement this interpretation. In the paste and molten states, loops became increasingly inclined with strain, indicating enhanced viscous dissipation and structural rearrangement. The enclosed area of the loops, which is proportional to energy loss per cycle, was greatest for SPC_0 and least for SPC_2%, confirming that oil addition mitigates internal friction (Figure 7). Conversely, in the weak-gel state, the larger loops for higher-oil samples reflected more complex viscoelasticity arising from the coexistence of a protein network and a partially structured oil phase.

Overall, the Lissajous analysis demonstrates that coconut-oil addition modulates the nonlinear viscoelasticity of SPC mixtures in a state-dependent manner: it weakens elasticity in the paste and melt but enhances rigidity in the weak-gel phase through thermally induced co-structuring of the dispersed oil and protein network.

### 2.4. Analysis of Chebyshev Coefficients for SPC Mixture with Different Oil Concentration

**Figure 8:**
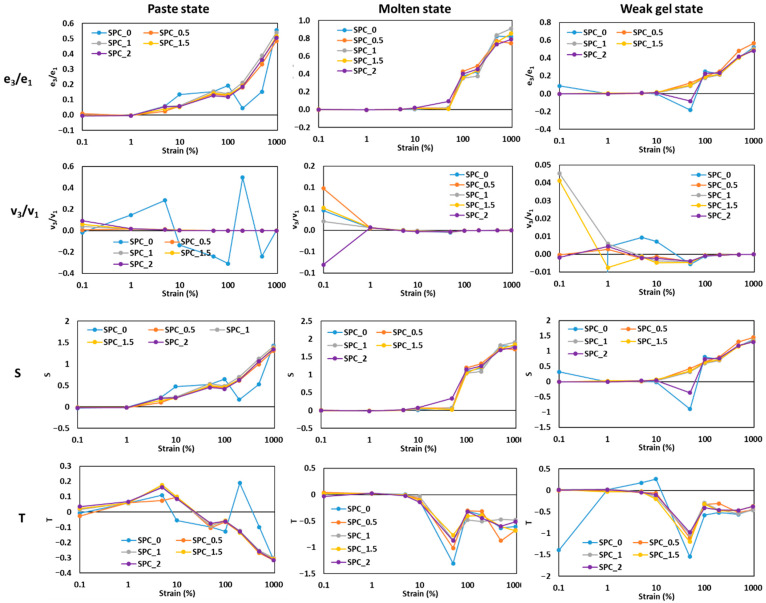
The changes in e_3_/e_1_, v_3_/v_1_, S, and T with respect to strain, γ (%), of SPC with different oil content at paste, molten, and weak gel state.

The Chebyshev decomposition (elastic e_3_/e_1_ and viscous v_3_/v_1_ coefficients) and derived metrics, i.e., the strain-stiffening ratio S and thickening ratio T, reveal how SPC nonlinear responses evolve with strain, oil content and thermal state (Figure 8). Interpreting these coefficients follows the framework of Ewoldt et al. (2007): e_3_/e_1_ > 0 signals intracycle strain-stiffening, e_3_/e_1_ < 0 indicates strain-softening; v_3_/v_1_ > 0 indicates intracycle shear-thickening, while negative values indicate shear-thinning [31].

In the paste state, S and e_3_/e_1_ are close to zero at very small strains (0.1–1%), consistent with linear response, then increased moderately at strains 5–100%, indicating increasing strain-stiffening as network segments stretch and align (Figure 8). For example, S increased markedly between 1% and 100% for SPC_0 and SPC_1, showing that unfilled SPC develops nonlinear elastic resistance when deformed large enough to engage protein–protein contacts. However, higher oil levels (1.5–2%) generally indicated a lower magnitude of S at intermediate strains, and v_3_/v_1_ tends toward smaller (often negative) values, evidence that oil acts to both blunt strain-stiffening and promote intracycle shear-thinning in the paste by lubricating interchain motions and lowering effective connectivity. These paste-state trends mirror LAOS findings in other food colloids where plasticizers or inclusions reduce nonlinear elastic signatures [32].

In the molten state, S values become substantially larger at high strains (≥100%), with e_3_/e_1_ and S both increasing strongly, particularly beyond 100–200% where S approaches and exceeds unity for some samples. This indicates pronounced strain-stiffening (elastic domination under large deformation) as thermally unfolded protein chains entangle and resist further stretch. Oil reduces the absolute K (SAOS) measures earlier, but in LAOS, the presence of modest oil does not fully suppress the high-strain elastic nonlinearities; instead, the oil slightly modulates the strain at which strong stiffening appears (shifting critical strain). Viscous coefficients v_3_/v_1_ in the molten state are small or slightly negative at intermediate strains, indicating net intracycle shear-thinning even as e_3_/e_1_ increases positively; this combination is typical of polymer-like, entangled melts undergoing LAOS [33].

In the weak-gel state, the coefficients show a distinct behavior: S and e_3_/e_1_ were positive across moderate-to-high strains and often reach large values earlier than in the molten state, while v_3_/v_1_ is small (near zero) or slightly negative at mid to high strains. This pattern implies the cooled gel supports large elastic stresses (strain-stiffening) and dissipates less per cycle relative to the paste. Notably, S increases with oil content up to 1.5% and then plateaus, consistent with the droplet-reinforcement hypothesis: during cooling, protein adsorption to oil interfaces and droplet–matrix coupling convert droplets into active fillers that augment intracycle elastic resistance. The plateau suggests interfacial saturation or crowding limits further reinforcement. These observations align with applied LAOS studies of emulsion-filled gels, where dispersed droplets increase elastic nonlinearities and reduce viscous dissipation once integrated into the network [34].

Overall, the Chebyshev analysis demonstrates a clear state-dependent behavior: the addition of oil reduces nonlinear elastic resistance during the molten or flowable stage, facilitating smoother flow and extrusion, but enhances nonlinear elasticity after cooling, contributing to a stronger, more cohesive gel network and improved textural stability.

### 2.5. CFD Simulation and Validation

**Figure 9:**
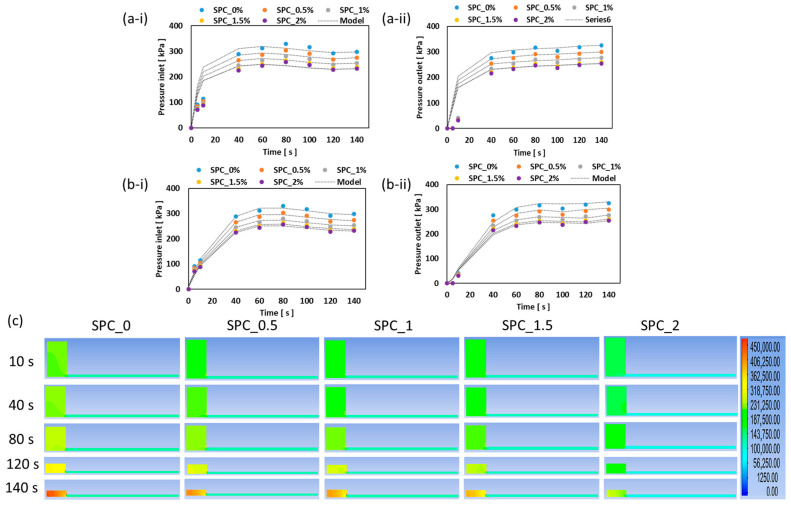
Pressure validation; (**a-i**,**a-ii**) measured vs. simulated pressure at the die inlet and outlet using the SAOS-based viscosity model, (**b-i**,**b-ii**) measured vs. simulated pressure at the die inlet and outlet using the LAOS-based viscoelastic model, and (**c**) LAOS-based simulation pressure profile along the extrusion chamber.

The results of the computational fluid dynamics (CFD) simulations demonstrate how effectively two rheological models, i.e., SAOS-based generalized viscosity model and a LAOS-based viscoelastic constitutive model, capture the observed extrusion behavior SPC melt with different oil concentration in the cooling die. As shown in Figure 9, the measured pressure at both the inlet and outlet decreases with increasing oil content across all tested oil concentrations, with an insignificant difference between SPC1.5% and SPC2% samples. Notably, the LAOS model yields an RMSE of ≤4.41 kPa, significantly outperforming the SAOS model, which has an RMSE value of ≤66.09 kPa. The poor performance of the SAOS model is attributed to its foundation in small-amplitude oscillatory rheology, which does not account for the elastic memory, strain-dependent structure, and temperature-driven gel stiffening that occur during cooling in the die. Conversely, the LAOS model captures nonlinear elasticity, shear-thinning-to-stiffening transitions, and the temperature-dependent relaxation spectrum, which become critical as the melt solidifies. This aligns with recent reviews that highlight LAOS analyses as more suitable for processing-relevant rheology of structured foods. For instance, Joyner [35] discussed how LAOS can be utilized to better understand the behavior of food materials under high shear processing conditions.

**Figure 10:**
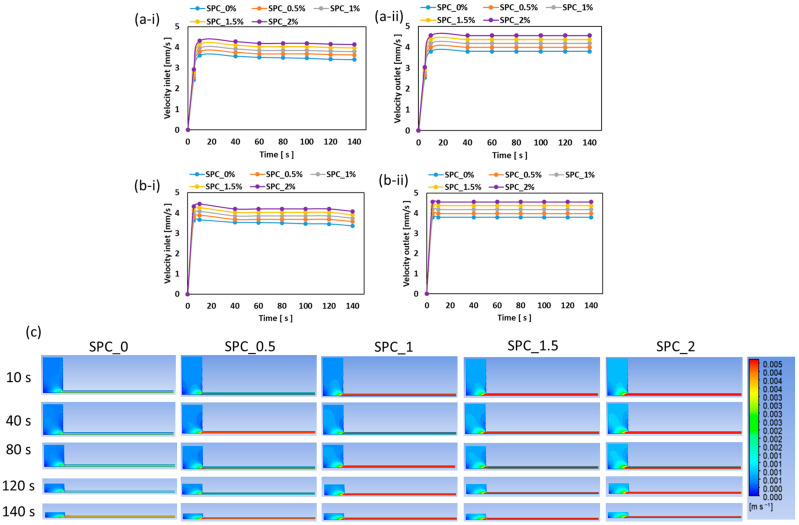
Velocity validation: (**a-i**,**a-ii**) simulated velocity at the die inlet and outlet using the SAOS-based viscosity model, (**b-i**,**b-ii**) simulated velocity at the die inlet and outlet using the LAOS-based viscoelastic model, and (**c**) velocity profile for LAOS based model.

The velocity validation results, presented in Figure 10, indicate that both outlet volumetric flow and local point velocities increased with oil content, which is consistent with oil reducing effective viscosity and wall friction. The LAOS model achieved an RMSE ≤ 0.11 mm/s compared to the SAOS-based model, which had an RMSE value of 0.33 mm/s. The improved velocity match with LAOS arises because it encodes strain- and temperature-dependent elastic resistance, which dominates near pinch-points or boundary-layer flow; a phenomenon often neglected in simple viscosity models but important in extrusion/die flow. McClements [36] has also emphasized the importance of understanding how food materials behave under various processing conditions, including extrusion, where complex rheological phenomena occur.

**Figure 11:**
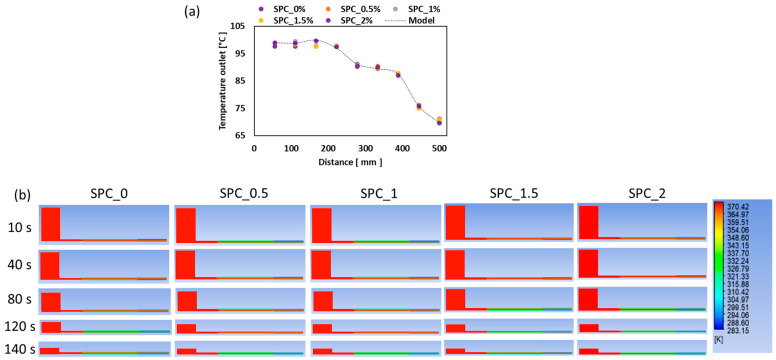
Temperature validation and sectional cooling: (**a**) measured vs. simulated temperature along the cooling die showing the three die temperature zones (100 °C, 50 °C, 10 °C), and (**b**) temperatures profile across the extrusion chamber.

Figure 11 illustrates the temperature distribution prediction and validation. Both models produced comparable results, with an RMSE of 0.57 °C at the die inlet and an RMSE of 0.75 °C at the die outlet. The temperature profile further shows a sectional cooling effect within the cooling die. At the cooling die inlet region, an average temperature of 99 °C was recorded, while the mid-section experienced a gradual decrease in melt temperature to an average value of 86 ± 1.32 °C due to the cooling effect at the die wall (50 °C wall). Similarly, the exit region (Section 3) indicated an average melt temperature of 70.3 ± 1.55 °C. The limited sensitivity to oil concentration or model choice is possibly due to heat transfer being dominated by boundary conditions (die wall temperatures set at 100, 50, and 10 °C, respectively, at each section of the cooling die), and the small oil fraction having minimal thermal-property contrast. Such observations reflect the common finding in food extrusion studies that low-fraction lipid additions influence mechanics more than heat transfer.

Overall, these results demonstrate that accurate prediction of pressure and velocity in cooling-die extrusion of SPC requires a rheological model capable of capturing structural evolution under shear, temperature drop, and gelation. Under these conditions, an SAOS-only model may be insufficient. The LAOS-informed viscoelastic model offers the fidelity needed for die-design and process optimization, though it comes with greater complexity and parameterization demands. For practical engineering trade-offs, an SAOS model may suffice for first-order estimates, but for detailed pressure/velocity prediction in gelation systems, the additional modeling effort is justified.

## 3. Conclusions

This study integrated nonlinear rheology (SAOS/LAOS) and computational fluid dynamics (CFD) to investigate the influence of coconut oil concentration (0–2%) on the thermal, rheological, and flow behavior of soy protein concentrate (SPC) mixtures during extrusion cooling. The findings provide a quantitative and mechanistic understanding of how oil alters viscoelasticity and gelation kinetics, with key implications for designing plant-based meat analogs. Dynamic temperature sweep tests revealed that increasing oil concentration delayed gelation, shifting the onset and peak temperatures from 64.13 °C and 70.29 °C (SPC_0%) to 70.21 °C and 76.08 °C (SPC_2%), while decreasing gelation enthalpy from 4.05 J/g to 2.81 J/g. This suggests that oil hinders protein aggregation, creating a softer, more lubricated matrix. LAOS analysis confirmed this trend, showing a transition from strain-stiffening in oil-free paste to strain-thinning in high-oil mixtures, reflecting easier deformation and structural breakdown at large strains. CFD simulations based on LAOS-derived constitutive parameters accurately predicted the pressure (RMSE values ≤ 4.41 kPa), velocity (RMSE ≤ 0.11 mm/s), and temperature profile (RMSE ≤ 0.75 °C) within the cooling die, significantly outperforming the SAOS-based model. This confirmed that incorporating nonlinear viscoelastic data is essential for accurately capturing extrusion flow behavior. The combined rheological–numerical framework provides a robust predictive tool for optimizing processing conditions and tailoring texture in oil-enriched SPC-based food products. Specifically, this approach enables engineers to select the necessary die cooling temperatures and residence times to ensure optimal gelation for any given oil concentration, thus facilitating rapid process design and formulation optimization. While this study provides a powerful predictive framework, it is limited to simulating flow only within the cooling die and focuses solely on saturated coconut oil. Future work should extend the CFD modeling to simulate the entire feeding zone, extruder screw, and barrel profile, accounting for complex mixing and plasticization zones. Furthermore, investigating the effect of unsaturated (liquid) oils, which have different phase behaviors and lower melting points, is necessary to generalize the model’s applicability across various commercial formulations.

## 4. Materials and Methods

### 4.1. Sample Preparation

The soy protein concentrate (SPC) used in this study was WDFSPC 690, produced from non-GMO defatted soy flakes through sequential processes of extraction, acidification, separation, neutralization, sterilization, drying, and packaging (Sujis Link Co., Ltd., Paju, Republic of Korea). In this process, defatted soy flakes are extracted with aqueous solvent to remove soluble carbohydrates and non-protein components. The extract is then acidified to precipitate proteins, separated, neutralized, sterilized, spray-dried to a stable powder, and packaged for storage. These steps remove anti-nutritional factors, yielding a protein-rich material suitable for food formulation.

According to the manufacturer’s specification sheet, WDFSPC 690 has a minimum protein content of 69% (dry basis) and a maximum moisture content of 8%. Its functional properties and high protein concentration make it suitable for use as a structural matrix in high-moisture extrusion of alternative meat products.

For sample preparation, SPC was mixed with distilled water and other minor ingredients commonly used in the plant-protein formulations to enhance water binding, texture, and color stability (Table 4). The selection and proportions of these minor ingredients are based on a proprietary, commercially relevant formulation used by Sujis Link Co., Ltd. for high-moisture extrusion. The mixture compositions were adjusted to obtain different oil inclusion levels (0–2% *w*/*w* coconut oil) while maintaining a constant overall moisture content of 80% across all samples. In all formulations, the proportions of Saylock mix (1.5%), baking powder (1%), and nucleic acid (0.04%) were kept constant to isolate the effect of oil content on the rheological behavior of the soy-protein system. The resulting mixtures were homogenized thoroughly to form uniform pastes prior to rheological and extrusion analyses.

### 4.2. Rheological Analysis and Gelation Kinetics

#### 4.2.1. Small Amplitude Oscillatory Shear (SAOS) Measurement

Dynamic oscillatory tests were performed using a Discovery Hybrid Rheometer HR-3 (TA Instruments, New Castle, DE, USA) equipped with a 40 mm parallel plate and Peltier temperature control system. The gap between plates was maintained at 2 mm, and all tests were conducted under controlled strain and temperature conditions. The SPC samples were examined under three structural states representative of the extrusion process: (i) paste, measured at room temperature (25 °C), (ii) molten melt, at 100 °C, and (iii) weak gel, tested after cooling to 70 °C. Before each measurement, the samples were allowed to rest for 5 min to ensure structural relaxation and uniform temperature distribution.

The viscosity test was first carried out over a shear rate range of 0.1–100 s^−1^ to evaluate shear-thinning behavior. Subsequently, a strain sweep (0.01–1000%) at a constant frequency of 1 Hz was performed to determine the linear viscoelastic region (LVR), where the storage modulus (G′) and loss modulus (G″) remained independent of strain. Within the LVR, a frequency sweep (0.1–100 rad s^−1^) was conducted at a constant strain of 0.1% to investigate the viscoelastic response of the SPC samples. This range was specifically chosen because it corresponds to the typical range of shear rates encountered during the extrusion process, simulating low-frequency, long-time deformations (e.g., in the cooling die) up to high-frequency, short-time deformations (e.g., at the die exit). The frequency dependence of G′ and G″ was described using the power-law model [11]:

(1) $$G^{\prime }=k^{\prime }.\omega^{n^{\prime }}$$

(2) $$G^{\prime \prime }=k^{\prime \prime }.\omega^{n^{\prime \prime }}$$

where k′ and k″ are consistency coefficients (Pa·s^n^) and n′ and n″ represent dimensionless frequency exponents.

To monitor gelation kinetics, a temperature sweep was performed from 20 to 100 °C at a heating rate of 5 °C min^−1^, followed by cooling to 25 °C, while continuously recording G′ and G″. These measurements provided insights into the thermal behavior and structure development of the soy protein system under different oil concentrations and thermal conditions.

#### 4.2.2. Large-Amplitude Oscillatory Shear (LAOS) Measurements

To evaluate the nonlinear viscoelastic properties of SPC at different structural states, Large-Amplitude Oscillatory Shear (LAOS) tests were performed using the Discovery Hybrid Rheometer HR-3 (TA Instruments, New Castle, DE, USA) under the same plate configuration as in the SAOS tests. The measurements were conducted for the paste (25 °C), molten melt (100 °C), and weak gel (75 °C) conditions. Before testing, each sample was allowed to relax for 5 min on the Peltier plate to eliminate residual stresses and achieve temperature equilibrium.

Oscillatory strain amplitude sweeps were applied over a range of 0.1–1000% strain at a constant angular frequency of 1 rad s^−1^. The critical strain (γ_cr_), i.e., the strain at which the storage modulus (G′) decreased by 5% from its linear viscoelastic plateau, was identified for each sample. The corresponding storage modulus (G′_cr_) at γ_cr_ was then used to calculate the cohesive energy density (E_c_), which represents the intermolecular energy required to deform the network [37]:

(3) $$E_{c}=\frac{1}{2}{\gamma_{cr}}^{2}{G^{\prime }}_{cr}$$

To characterize the nonlinear viscoelastic response in more detail, the MITlaos software (Version 2.1 Beta), incorporating the nonlinear analytical framework proposed by Ewoldt et al. [31], was applied to interpret the Lissajous curves and extract the discrete Chebyshev coefficients from the stress–strain data. These coefficients describe the harmonic contributions of the material’s response, where e_1_, e_3_, e_5_,… correspond to the elastic harmonics, and v_1_, v_3_, v_5_,… represent the viscous harmonics. The ratios e_3_/e_1_ and v_3_/v_1_ quantify the relative magnitude of nonlinear effects in the elastic and viscous domains, respectively, and were used to characterize the rheological behavior of the soy protein system under large deformation. Within the linear viscoelastic regime, the influence of higher-order harmonics is negligible, yielding e_3_ = 0 and v_3_ = 0. A positive e_3_/e_1_ value signifies intra-cycle strain stiffening, while a negative ratio denotes strain softening. Likewise, v_3_/v_1_ > 0 indicates shear thickening, whereas v_3_/v_1_ < 0 corresponds to shear thinning.

Additional nonlinear viscoelastic parameters describing the SPC structure were derived from the Chebyshev coefficients, as defined in Equations (4)–(9) [29]:

(4) $${G^{\prime }}_{M}={\left.\frac{d\sigma }{d\gamma }\right|}_{\gamma =0}\approx e_{1}-{3e}_{3}+{5e}_{5}-{7e}_{7}+\cdots ,$$

(5) $${G^{\prime }}_{L}={\left.\frac{\sigma }{\gamma }\right|}_{\gamma =\pm \gamma_{0}}\approx e_{1}+e_{3}+e_{5}+e_{7}+\cdots ,$$

where e_1_, e_3_, e_5_, … denote the elastic Chebyshev coefficients previously defined. Consequently, G′_M_ represents the instantaneous modulus at γ = 0 (the minimum strain or maximum shear rate), whereas G′_L_ corresponds to the modulus at γ = ±γ_0_ (the peak applied strain).

Similarly, the nonlinear viscous parameters were calculated at the smallest and largest resolvable shear rates according to Equations (6) and (7) [38,39]:

(6) $${\eta^{\prime }}_{M}={\left.\frac{d\sigma }{d\dot{\gamma }}\right|}_{\dot{\gamma }=0}\approx v_{1}-{3v}_{3}+{5v}_{5}-{7v}_{7}+\cdots ,$$

(7) $${\eta^{\prime }}_{L}={\left.\frac{\sigma }{\dot{\gamma }}\right|}_{\dot{\gamma }=\pm {\dot{\gamma }}_{0}}\approx v_{1}+v_{3}+v_{5}+v_{7}+\cdots ,$$

To further evaluate intra-cycle nonlinearities, two dimensionless ratios, i.e., the stiffening ratio (S) and thickening ratio (T) were obtained as [37]:

(8) $$S=\frac{{G^{\prime }}_{L}-{G^{\prime }}_{M}}{{G^{\prime }}_{L}}\approx \frac{{4e}_{3}-{4e}_{5}+{8e}_{7}+\cdots }{e_{1}+e_{3}+e_{5}+e_{7}+\cdots }$$

(9) $$T=\frac{{\eta^{\prime }}_{L}-{\eta^{\prime }}_{M}}{{\eta^{\prime }}_{L}}\approx \frac{{4v}_{3}-{4v}_{5}+{8v}_{7}+\cdots }{v_{1}+v_{3}+v_{5}+v_{7}+\cdots }$$

Both parameters effectively describe the nonlinear deformation behavior of viscoelastic systems: positive S values indicate strain stiffening, negative S values denote strain softening, while positive T values correspond to shear thickening and negative T values to shear thinning [31].

#### 4.2.3. Thermal Analysis of SPC Paste

The thermal transitions of the SPC mixtures were examined using a Discovery Series Differential Scanning Calorimeter (DSC) (TA Instruments, New Castle, DE, USA). Approximately 10–15 mg of each homogenized sample was weighed with an accuracy of ±0.01 mg and hermetically sealed in stainless-steel DSC pans to prevent moisture loss during heating. A sealed, empty pan served as the reference [4].

Thermal scans were carried out from 20 °C to 100 °C under a constant heating rate of 5 °C/min, while a nitrogen gas purge was maintained at 50 mL/min to ensure an inert environment and prevent oxidative reactions [4]. This rate was chosen based on preliminary experiments that maximized the resolution of the thermal events (gelation onset, peak, and endset) while maintaining a relevant time scale for the processing conditions. The resulting thermograms were recorded and analyzed using Trios software (v5.0.0, TA Instruments, New Castle, DE, USA) to determine the onset temperature (T_o_), peak denaturation temperature (T_p_), and end-set temperature (T_e_) of protein transitions. The enthalpy change (ΔH), expressed in J/g of protein, was obtained by integrating the area under the endothermic peak corresponding to the protein denaturation event. All analyses were performed in triplicate to ensure reproducibility, and the thermal parameters were used to evaluate how increasing coconut oil concentration influenced the thermal stability and gelation behavior of the SPC matrix.

### 4.3. Extrusion Process

**Figure 12:**
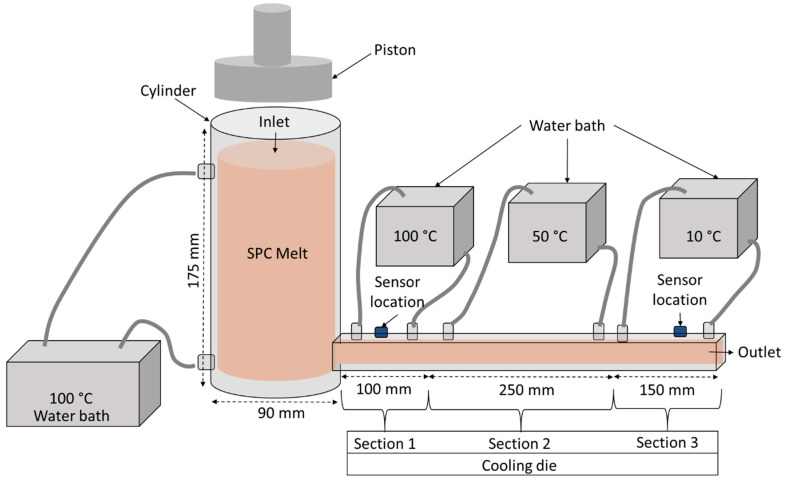
Schematic representation of experimental setup for SPC extrusion. Variable process parameters were the concentration of oil (0, 0.5, 1, 1.5, and 2%), and melt cooling stages.

The extrusion of the SPC mixtures was performed using a custom-fabricated single-barrel extrusion system designed to simulate the heating, compression, and cooling stages of HME (Figure 12). The extrusion barrel, made from stainless steel (SUS 304, POSCO Co., Ltd., Pohang, Republic of Korea), had an internal diameter of 90 mm and a height of 175 mm, ensuring corrosion resistance and uniform thermal conduction under high-moisture and high-temperature conditions.

To achieve controlled heating, two hollow channels were machined around the barrel wall for continuous hot-water circulation supplied from a thermostatic water bath (Model RW-3025G, Jeiotech Co., Daejeon, Republic of Korea). The water bath maintained a stable heating environment (100 °C) for 60 min, ensuring complete melting and equilibration of the protein–oil–water mixture. Compression of the molten mass was performed using a texture analyzer (TA.XT Plus, Stable Micro Systems, Surrey, UK) equipped with an 89.8 mm piston, operating at a constant speed of 1 mm/s to generate pressure comparable to shear and compression in industrial extrusion zones.

Following compression, the molten SPC was extruded through a rectangular cooling die (20 mm × 10 mm cross-section, and 500 mm in total length). The cooling die was divided into three temperature-controlled sections to mimic the progressive solidification occurring during real HME. The first section (0–100 mm from the barrel outlet) was maintained at 100 °C, allowing the melt to begin alignment of protein strands while remaining in a fluid-like state. The second section (>100–350 mm along the die length) was surrounded by circulating water maintained at 50 °C, promoting gradual cooling and partial network formation. The final section (>350 mm) was cooled rapidly using chilled water at 10 °C, inducing solidification and stabilizing the structure into a weak gel form. The outlet temperature of the extrudate was 71 ± 1.35 °C, consistent with the transition to a weakly elastic gel.

Temperature and pressure of the melt were continuously monitored at two points: (i) 5 cm from the point where the melt entered the cooling die (tagged Die Inlet) and (ii) 5 cm before exiting the die (tagged Die Exit) (Figure 12). For temperature measurement, K-type thermocouples (MAX6675 module, Adafruit Industries, New York, NY, USA) were embedded flush with the die wall and connected to an Arduino Uno for data acquisition. Pressure was recorded using stainless-steel piezoresistive transducers (Model XGZP6847, CFSensor, Wuhu, China) compatible with Arduino, capable of operating up to 150 °C. Both sensors were sealed in threaded ports and thermally insulated to prevent flow disturbance or local heat loss.

This configuration enabled real-time monitoring of pressure and temperature evolution within the cooling die, allowing accurate validation of the CFD model. After extrusion, the weak gel samples were collected and sealed in polyethylene films to prevent moisture loss prior to rheological and structural analysis.

### 4.4. Computational Fluid Dynamics (CFD) Simulation of the Extrusion Flow

Computational simulations of the extrusion flow and thermal behavior of SPC mixtures were conducted using ANSYS Fluent 2024 R2 (ANSYS Inc., Canonsburg, PA, USA). Two constitutive modeling strategies were implemented and compared: Model A, a generalized Newtonian model based on SAOS-derived viscosity data, and Model B, a viscoelastic model of the Phan-Thien–Tanner (PTT) type informed by LAOS nonlinear behavior [40]. The aim was to assess how well each model predicts key process variables (pressure drop, velocity profile, temperature profile) as oil concentration changes.

#### 4.4.1. Governing Equations and Constitutive Models

The flow field was governed by the incompressible continuity and momentum equations:

(10) ∇·u=0

(11) $$\rho \left(\frac{\partial u}{\partial t}+u\cdot \nabla u\right)=-\nabla p+\nabla \cdot \tau +pg$$

Simultaneously, the energy equation was solved to account for thermal effects and viscous dissipation:

(12) $$\rho C_{p}\left(\frac{\partial T}{\partial t}+u\cdot \nabla T\right)=\nabla \cdot \left(k\nabla T\right)+\Phi$$

where ρ is the density (kg m^−3^), u the velocity vector (m s^−1^), p the pressure (Pa), τ the extra stress tensor (Pa), g the gravitational acceleration (m s^−2^) cp is the specific heat (J kg^−1^ K^−1^), k is thermal conductivity (W m^−1^ K^−1^), T is temperature (K), and Φ is the viscous dissipation term (W m^−3^) [6].

In the SAOS-based model (Model A), the extra stress tensor was approximated by a generalized Newtonian form:

(13) $$\tau =2\eta_{app}\left(\dot{\gamma },\;T\right)D$$

where $D=\frac{1}{2}(\nabla u+\nabla u^{T})$ is the rate-of-deformation tensor and $\dot{\gamma }=\sqrt{2D:D}$ is the scalar shear rate (s^−1^). The apparent viscosity $\eta_{app}\left(\dot{\gamma },\;T\right)$ defined according to a power-law model fitted to frequency sweep, is expressed as:

(14) $$\eta_{app}\left(\dot{\gamma },\;T\right)=K(T){\dot{\gamma }}^{n\left(T\right)-1}$$

The temperature dependence of the consistency index K(T) was expressed using an Arrhenius-type relation:

(15) $$K(T)=K_{0}exp\left(\frac{E_{a,k}}{R}\left(\frac{1}{T}-\frac{1}{T_{ref}}\right)\right)$$

(16) $$n\left(T\right)=n_{0}+\alpha_{n}\left(T-T_{ref}\right)$$

where K_0_ is the consistency coefficient at reference temperature T_ref_ (k), E_a,k_ is the activation energy (J mol^−1^), R is the universal gas constant, n_0_ is the flow behavior exponent at T_ref_, and α_n_ is the temperature sensitivity coefficient of n. This explicit form ensures viscosity varies with both shear rate and temperature, appropriate for the cooling die profile experienced in extrusion [8].

In the LAOS-based model (Model B), the material’s viscoelastic behavior was captured via a differential constitutive equation of the Phan-Thien-Tanner (PTT) type [40]:

(17) $$\tau +\lambda (T)f(\tau )\begin{array}{c} \nabla \\ \tau \end{array}=2\eta_{0}(T)D$$

where τ is the extra stress tensor (Pa), $D=\frac{1}{2}(\nabla u+\nabla u^{T})$ is the rate-of-deformation tensor, λ(T) is the temperature-dependent relaxation time (s), $\eta_{0}$ (T) is the zero-shear viscosity (Pa·s), and f(τ) is a damping function representing the finite extensibility of the viscoelastic network (dimensionless). Previous studies on viscoelastic melts have shown that PTT models effectively describe the extrusion behavior of non-Newtonian melts, providing better numerical stability for biopolymer systems [41].

(18) $$f\left(\tau \right)=exp\left[\epsilon \frac{\lambda \left(T\right)}{\eta_{0}\left(T\right)}tr(\tau )\right]$$

where ϵ (dimensionless) is the damping coefficient controlling the extent of nonlinearity. All temperature dependencies were described using Arrhenius-type relations:

(19) $$\eta_{0}(T)=\eta_{0,ref}exp\left(\frac{E_{\eta }}{R}\left(\frac{1}{T}-\frac{1}{T_{ref}}\right)\right)$$

(20) $$\lambda \left(T\right)=\lambda_{ref}exp\left(\frac{E_{\lambda }}{R}\left(\frac{1}{T}-\frac{1}{T_{ref}}\right)\right)$$

where E_η_ and E_λ_ are activation energies for viscosity and relaxation time, respectively.

Parameters $\eta_{0}$ λ, and ϵ, were extracted from the LAOS data using MITlaos software (MITlaos Ver. 2.1 Beta), based on: (i) relaxation time (λ) from crossover frequency wc=1λ, (ii) zero-shear viscosity ($\eta_{0}$) from low-frequency plateau modulus (${G^{\prime }}_{0})$ as $\eta_{0}={G^{\prime }}_{0}\lambda$, and (iii) damping coefficient (ϵ) derived from harmonic ratios e_3_/e_1_ and v_3_/v_1_. These parameters were then entered in Fluent through a User-Defined Rheology (UDRAN) subroutine, which solves Equations (17) and (18) locally at each cell and time step using the Finite Volume Method (FVM).

The UDRAN routine was compiled in C language and loaded through the Fluent Dynamic Library. At each iteration, the code updated the stress tensor and viscosity fields as functions of local temperature and shear rate.

#### 4.4.2. Simulation Domain, Boundary Conditions and Material Inputs

The physical geometry mirrored the experimental extrusion setup (Section 2.3): a cylindrical barrel (D = 90 mm, H = 175 mm) feeding a rectangular cooling die (20 mm × 10 mm, L = 500 mm). A structured finite-volume mesh with approximately 1.2 million cells was generated, with refinement near wall boundaries and the barrel–die transition to ensure shear gradients were well resolved. Mesh independence was confirmed by verifying that outlet pressure changed by less than 2% when cell count was increased.

Boundary conditions included a volumetric flow rate corresponding to the 1 mm/s piston speed (Section 2.3) at the inlet, a no-slip condition at the walls, barrel and die walls assigned prescribed temperature zones of 100 °C (barrel/melt), 50 °C (mid die; Section 2), and 10 °C (end die; Section 3), and a zero gauge pressure at the outlet (Figure 12). Energy equations were solved to account for viscous dissipation and heat transfer (k, c_p_ and ρ defined for SPC blends, Table 5).

#### 4.4.3. CFD Simulation Strategy and Model Calibration

Simulations were conducted under steady-state conditions for each oil level (0%, 0.5%, 1%, 1.5%, 2.0%) and structural state (paste, melt, weak gel). The computational flow field was modeled using three primary physical assumptions applied to the governing Equations (10)–(12). Firstly, the flow was considered steady-state (time-independent). Secondly, the flow was assumed to be laminar, justified by the high viscosity of the SPC mixtures and the low piston velocity, resulting in a low Reynolds number. Lastly, the simulation was treated as non-isothermal because the energy equation was solved simultaneously, thereby accounting for temperature-dependent viscosity and the internal heat generation from viscous dissipation, which is critical for accurate prediction of the cooling die profile. Model performance was assessed via root-mean-square error (RMSE) between predicted and experimental values for pressure, temperature, and velocity:

(21) $${RMSE}_{pressure}=\sqrt{\frac{1}{N}\sum_{i=1}^{N}{\left({Pressure}_{sim,i}-{Pressure}_{exp,i}\right)}^{2}}$$

(22) $${RMSE}_{Temperature}=\sqrt{\frac{1}{N}\sum_{i=1}^{N}{\left(T_{sim,i}-T_{exp,i}\right)}^{2}}$$

(23) $${RMSE}_{velocity}=\sqrt{\frac{1}{N}\sum_{i=1}^{N}{\left({Velocity}_{sim,i}-{Velocity}_{exp,i}\right)}^{2}}$$

Convergence criteria were based on continuity residual <10^−6^, momentum and energy residuals <10^−5^. Good agreement was defined as prediction errors within ±5 % of the experimental readings.

#### 4.4.4. Statistical Analysis

All experimental data (rheology and DSC) were reported as the mean ± standard deviation of triplicate measurements. The effect of coconut oil concentration on the measured parameters was analyzed using a One-Way Analysis of Variance (ANOVA). Following the ANOVA, Tukey’s Honest Significant Difference (HSD) post hoc test was performed to determine significant differences between the means of the various oil concentration levels. Statistical significance was defined at a confidence level of *p* < 0.05. Statistical computations were performed using IBM SPSS Statistics (version 27, IBM Corporation, Armonk, New York, NY, USA).

## Figures and Tables

**Table 1:** Crossover strain (%), critical strain (*γ*_*c*r_), the corresponding storage modulus (*G*′_*cr*_) at critical strain, and the cohesive energy density of SPC mixture at different oil concentrations and heating stages.

	Sample	Crossover Strain (%)	Critical Strain, *γ*_*c*r_ (%)	G′ at Critical Strain, G′_cr_ (Pa)	Cohesive Energy Density, E_c_ (J/m^3^)
	SPC_0	Nil	0.27 ± 0.05 ^a^	77.09 ± 1.14 ^a^	2.72 ± 0.04 ^a^
Paste state	SPC_0.5	Nil	0.23 ± 0.03 ^a^	56.53 ± 2.35 ^b^	1.48 ± 0.03 ^b^
	SPC_1	Nil	0.19 ± 0.01 ^b^	54.79 ± 4.11 ^c^	1.01 ± 0.11 ^c^
	SPC_1.5	Nil	0.18 ± 0.02 ^b^	33.52 ± 2.19 ^d^	0.52 ± 0.08 ^d^
	SPC_2	Nil	0.16 ± 0.02 ^bc^	23.94 ± 1.96 ^e^	0.32 ± 0.06 ^e^
	SPC_0	3.17 ± 0.26 ^a^	0.16 ± 0.03 ^a^	8981.64 ± 276.49 ^d^	117.92 ± 5.39 ^a^
Molten state	SPC_0.5	1.61 ± 0.31 ^a^	0.16 ± 0.02 ^a^	6014.39 ± 199.64 ^e^	73.94 ± 9.46 ^b^
	SPC_1	1.53 ± 0.09 ^a^	0.15 ± 0.02 ^a^	3791.64 ± 105.37 ^a^	43.54 ± 3.14 ^c^
	SPC_1.5	0.67 ± 0.15 ^a^	0.15 ± 0.04 ^ab^	2923.33 ± 164.88 ^b^	32.78 ± 5.23 ^d^
	SPC_2	0.67 ± 0.07 ^a^	0.15 ± 0.04 ^ab^	1843.51 ± 98.64 ^c^	20.37 ± 2.03 ^e^
	SPC_0	4.85 ± 0.45 ^a^	0.18 ± 0.03 ^bc^	9508.49 ± 328.44 ^d^	147.01 ± 7.65 ^d^
Weak gel state	SPC_0.5	3.17 ± 0.29 ^a^	0.19 ± 0.05 ^b^	14,626.74 ± 561.23 ^c^	262.77 ± 11.65 ^c^
	SPC_1	1.61 ± 0.07 ^a^	0.20 ± 0.04 ^b^	30,817.52 ± 615.18 ^b^	626.55 ± 45.77 ^b^
	SPC_1.5	1.60 ± 0.05 ^a^	0.31 ± 0.08 ^a^	36,514.65 ± 594.39 ^a^	1715.48 ± 189.64 ^a^
	SPC_2	1.59 ± 0.06 ^a^	0.29 ± 0.07 ^a^	36,499.80 ± 561.78 ^a^	1563.67 ± 167.28 ^a^

Values within the same column and similar heating stage of sample having different superscripts (a, b, c, d, and e) differ significantly (*p* < 0.05).

**Table 2:** Damping factor (G″/G′ at 1, 4, and 100 rad/s), consistency indices (K′, K″), and flow behavior exponents (n′, n″) of soy protein concentrate mixtures with varying oil concentrations in different structural states.

	Sample	Damping Factor (rad/s)			K′	n′	K″	n″
		1	4	100				
	SPC_0	3.522 ± 0.05 ^b^	4.125 ± 0.05 ^a^	5.023 ± 0.05 ^a^	679.3 ± 11.1 ^a^	0.139 ± 0.003 ^a^	2534 ± 22.4 ^a^	0.206 ± 0.005 ^a^
Paste state	SPC_0.5	3.740 ± 0.04 ^a^	4.023 ± 0.06 ^b^	4.740 ± 0.06 ^b^	613.5 ± 16.6 ^b^	0.140 ± 0.004 ^a^	2257 ± 25.1 ^b^	0.194 ± 0.006 ^b^
	SPC_1	3.309 ± 0.03 ^d^	3.771 ± 0.07 ^c^	4.406 ± 0.07 ^c^	551.1 ± 27.2 ^c^	0.129 ± 0.005 ^b^	1850 ± 28.4 ^c^	0.191 ± 0.006 ^b^
	SPC_1.5	3.447 ± 0.04 ^c^	3.765 ± 0.08 ^c^	4.121 ± 0.08 ^e^	481.9 ± 18.2 ^d^	0.124 ± 0.006 ^bc^	1697 ± 19.7 ^d^	0.173 ± 0.007 ^d^
	SPC_2	3.521 ± 0.06 ^b^	4.036 ± 0.09 ^b^	4.491 ± 0.09 ^d^	411.2 ± 19.3 ^e^	0.121 ± 0.007 ^c^	1436 ± 27.3 ^e^	0.186 ± 0.005 ^c^
Molten state	SPC_0	0.309 ± 0.03 ^d^	0.301 ± 0.03 ^b^	0.263 ± 0.03 ^b^	4071.0 ± 10.3 ^a^	0.138 ± 0.002 ^a^	1254 ± 12.7 ^a^	0.096 ± 0.005 ^a^
	SPC_0.5	0.315 ± 0.04 ^b^	0.298 ± 0.04 ^c^	0.262 ± 0.04 ^b^	3557.0 ± 15.7 ^b^	0.135 ± 0.002 ^a^	1111 ± 15.4 ^b^	0.097 ± 0.006 ^a^
	SPC_1	0.307 ± 0.05 ^d^	0.276 ± 0.05 ^d^	0.253 ± 0.05 ^c^	3258.0 ± 10.5 ^c^	0.129 ± 0.003 ^b^	978.7 ± 18.6 ^c^	0.085 ± 0.007 ^b^
	SPC_1.5	0.311 ± 0.06 ^bc^	0.310 ± 0.05 ^a^	0.282 ± 0.06 ^a^	2742.0 ± 15.3 ^d^	0.117 ± 0.001 ^c^	862.9 ± 22.6 ^d^	0.087 ± 0.008 ^b^
	SPC_2	0.336 ± 0.07 ^a^	0.298 ± 0.07 ^c^	0.285 ± 0.07 ^a^	2334.0 ± 13.2 ^e^	0.117 ± 0.001 ^c^	745.8 ± 25.1 ^e^	0.084 ± 0.009 ^b^
Weak gel state	SPC_0	0.305 ± 0.03 ^a^	0.338 ± 0.03 ^a^	0.371 ± 0.03 ^a^	4962.0 ± 9.1 ^e^	0.025 ± 0.002 ^b^	1598 ± 12.7 ^e^	0.050 ± 0.001 ^c^
	SPC_0.5	0.283 ± 0.04 ^b^	0.290 ± 0.04 ^b^	0.313 ± 0.04 ^b^	6714.0 ± 13.8 ^d^	0.019 ± 0.002 ^c^	1851 ± 17.5 ^d^	0.052 ± 0.001 ^c^
	SPC_1	0.237 ± 0.05 ^c^	0.240 ± 0.05 ^c^	0.257 ± 0.05 ^c^	8661.0 ± 9.5 ^c^	0.024 ± 0.001 ^b^	2007 ± 28.6 ^c^	0.056 ± 0.001 ^b^
	SPC_1.5	0.215 ± 0.06 ^d^	0.218 ± 0.06 ^d^	0.247 ± 0.06 ^cd^	10,970.0 ± 11.8 ^b^	0.026 ± 0.001 ^a^	2373 ± 21.2 ^db^	0.061 ± 0.001 ^a^
	SPC_2	0.219 ± 0.07 ^d^	0.220 ± 0.07 ^d^	0.238 ± 0.07 ^d^	12,641.0 ± 11.3 ^a^	0.028 ± 0.001 ^a^	2667 ± 29.9 ^a^	0.062 ± 0.001 ^a^

Values within the same column and sample state having different superscripts (a, b, c, d, and e) differ significantly (*p* < 0.05).

**Table 3:** Gelation thermal parameters of SPC mixtures containing 0–2% coconut oil: onset of gelation, peak gelation temperature, end of gelation and gelation enthalpy (J/g protein).

Sample	Onset of Gelation (°C)	Peak Gelation Temperature (°C)	End of Gelation (°C)	Gelation Enthalpy (J/g)
SPC_0	64.13 ± 0.41 ^e^	70.29 ± 1.25 ^d^	76.34 ± 1.31 ^c^	2.81 ± 0.64 ^d^
SPC_0.5	65.48 ± 0.37 ^d^	71.51 ± 1.03 ^c^	77.73 ± 1.22 ^bc^	3.10 ± 0.72 ^c^
SPC_1	67.11 ± 0.51 ^c^	73.28 ± 0.94 ^b^	79.57 ± 0.83 ^b^	4.02 ± 0.81 ^a^
SPC_1.5	68.39 ± 0.48 ^b^	74.66 ± 0.87 ^ab^	81.54 ± 1.68 ^a^	4.05 ± 0.664 ^a^
SPC_2	70.21 ± 0.62 ^a^	76.08 ± 1.11 ^a^	82.69 ± 1.32 ^a^	3.91 ± 0.77 ^ab^

Values within the same column having different superscripts (a, b, c, d, and e) differ significantly (*p* < 0.05).

**Table 4:** Mixture formulations of soy protein concentrate containing varying oil levels (0–2% *w*/*w*) at fixed moisture content (80%).

	Oil Concentration				
	SPC_0	SPC_0.5	SPC_1	SPC_1.5	SPC_2
Distilled water (mL)	78.45	78.49	78.54	78.58	78.62
Conc soy protein (%)	19.01	18.47	17.92	17.38	16.84
Saylock mix (%)	1.5	1.5	1.5	1.5	1.5
Baking powder (%)	1	1	1	1	1
Nucleic acid (%)	0.04	0.04	0.04	0.04	0.04
Coconut oil (%)	0	0.5	1	1.5	2
Total	100	100	100	100	100

SPC indicates soy protein concentrate, and the numerical number indicates the percentage concentration of coconut oil.

**Table 5:** Thermophysical and rheological parameter template.

Sample	Density, ρ (kg m^−3^)	Specific Heat, c_p_ (J kg^−1^ K^−1^)	Thermal Conductivity, k (W m^−1^ K^−1^)	Zero-Shear Viscosity, η_0_ (Pa·s)	Relaxation Time, λ (s)	Damping Coefficient (ϵ)
SPC_0	1052	4 × 10^−15^T^2^ + 0.362T + 3606	−9 × 10^−19^T^2^ − 0.0003T + 0.5265	5112	1.0	0.05
SPC_0.5	1049	8 × 10^−6^T^2^ + 0.3609T + 3599	2 × 10^−8^T^2^ − 0.0003T + 0.5163	4257	0.9	0.05
SPC_1	1047	0.361T + 3591	−0.0005T + 0.5125	3625	0.8	0.05
SPC_1.5	1045	4 × 10^−6^T^2^ + 0.3589T + 3581	−5 × 10^−8^T^2^ − 0.0005T + 0.5022	3011	0.7	0.05
SPC_2	1041	4 × 10^−6^T^2^ + 0.3586T + 3571	−4 × 10^−8^T^2^ − 0.0005T + 0.4922	2707	0.6	0.05

T represent the sample temperature.

## Data Availability

The data presented in this study are available in the article. Further inquiries can be directed to the corresponding author.
